# Profiling First-Year Students in STEM Programs Based on Autonomous Motivation and Academic Self-Concept and Relationship with Academic Achievement

**DOI:** 10.1371/journal.pone.0112489

**Published:** 2014-11-12

**Authors:** Carolien Van Soom, Vincent Donche

**Affiliations:** 1 Faculty of Science, Leuven Engineering and Science Education Center (LESEC), University of Leuven, Leuven, Belgium; 2 Institute of Education and Information Science, University of Antwerp, Antwerp, Belgium; University of Minho, Portugal

## Abstract

The low success rate of first-year college students in Science, Technology, Engineering, and Mathematics (STEM) programs has spurred many academic achievement studies in which explanatory factors are studied. In this study, we investigated from a person-oriented perspective whether different motivational and academic self-concept profiles could be discerned between male and female first-year college students in STEM and whether differences in early academic achievement were associated with these student groups. Data on autonomous motivation, academic self-concept, and early academic achievement of 1,400 first-year STEM college students were collected. Cluster analyses were used to distinguish motivational profiles based on the relative levels of autonomous motivation and academic self-concept for male and female students. Differences in early academic achievement of the various profiles were studied by means of ANCOVA. Four different motivational profiles were discerned based on the dimensions of autonomous motivation (A) and academic self-concept (S): students scoring high and respectively low on both dimensions (HA-HS or LA-LS), and students scoring high on one dimension and low on the other (HA-LS or LA-HS). Also gender differences were found in this study: male students with high levels of academic self-concept and autonomous motivation had higher academic achievement compared to male students with low levels on both motivational dimensions. For female students, motivational profiles were not associated with academic achievement. The findings partially confirm the internal and external validity of the motivational theories underpinning this study and extend the present insights on identifying subgroup(s) of at risk students in contemporary STEM programs at university level.

## Introduction

Even though there is a high market demand for Science, Technology, Engineering, and Mathematics (STEM) graduates, the combination of a declining interest of high school students for STEM studies and the low success rates of first-year college students remains problematic [1]. To better understand the STEM first-year experience and to identify targets for effective interventions, empirical research on academic achievement and retention in STEM programs is essential. STEM programs require a strong academic preparation in mathematics since mathematics modules of varying complexity are obligatory in most first-year STEM programs. As a result, numerous STEM retention studies focus on grades and mathematics test scores as explanatory factors of study success [2]–[4]. Besides cognitive factors, various motivational factors have been found to be important predictors of first-year academic achievement and study persistence [2], [3], [5], [6]. In a meta-analysis of psychological correlates of university students' academic performance in general, performance self-efficacy was found the strongest correlate, followed by high school GPA and ACT scores [7]. Besides academic self-concept [8], also autonomous motivation has been repeatedly associated with academic achievement [9]. It is remarkable that although autonomous motivation and academic self-concept have frequently been found to be important predictors for academic achievement, both constructs are often separately investigated by using variable-centered analysis in which variables or groups of variables form the unit of analysis and not individuals or subgroups of individuals [10]–[12]. Although most motivational theories agree on the multi-dimensional and complex nature of motivation, the intricate interplay of these variables within subgroups of respondents are scarcely investigated [13].

### Autonomous motivation, academic self-concept and academic achievement

According to self-determination theory (SDT), motivation is a multidimensional concept, in which different motivation types can be discerned that vary in their degree of self-determination [14]. The different motivation types range from a pure intrinsic motivation (for behavior that is inherently interesting or enjoyable), over identified regulation (behavior driven by a personally held goal or value), and introjected regulation (behavior spurred by internal pressure, to avoid guilt), to external regulation (behavior driven by external constraints) [14]. Autonomous motivation triggers behavior driven by pleasure or personal choice, and comprises both intrinsic and identified types of regulation, whereas controlled motivation triggers behavior driven by pressure, that can be external (avoid punishment) or internal (to avoid feelings of guilt), and comprises introjected and external motivational drives. SDT has been applied in research on student motivation and shown that different types of motivation have a differential impact on outcomes such as academic achievement of college students [14], [15]. In general, various variable-centered analyses with university student and junior college students have shown that in an autonomy supportive learning environment, autonomous motivation is positively associated with academic achievement, increased study persistence and decreased drop-out [16]–[18]

Academic self-concept is a subjective judgement of one's perceived ability in an academic or learning context [19], [20]. The multidimensional structure of self-concept has been the subject of much of debate, and distinctions have been made between general academic self-concept, which is a good predictor of general academic achievement measures such as GPA [21], and several domain-specific self-concepts (e.g. math or verbal self-concept), which are more closely related to specific course achievement [22], [23].

Since academic self-concept is positively associated with academic achievements [21], [24], many studies have focused on the directional or even causal link between academic self-concept and achievement [25], [26]. Three distinct models have been proposed to describe the relationship between academic self-concept and academic achievement: the skill development model (academic achievement determines academic self-concept), the self-enhancement model (academic self-concept determines achievement) and the reciprocal effects model (achievement and self-concept mutually reinforce each other) [27]. There is conflicting evidence on a developmental perspective in the causal order, where some studies report that during early school years (elementary school), the skill development model is predominant [28], and that only during middle school, ability perceptions become more stable so that the relationship between academic achievement and academic self-concept becomes reciprocal [29]. In the final years of high school, numerous studies have shown that prior academic self-concept predicts subsequent achievement, which in turn influences subsequent academic self-concept after controlling for prior achievement [8], [30], [31].

Longitudinal research in the final year of high school showed that students' academic self-concept was predictive for success in the first year of higher education, after controlling for high school achievement and gender [32]. It was found that female high school students reported a lower academic self-concept than male students, even when controlling for individual achievement [32]. Also in first year university STEM students, math and science self-concept of female students was lower compared to male students [33]. In a longitudinal study of first year university students in social science, there was no gender difference in academic self-concept at the beginning of higher education, but after the first semester at university, general academic self-concept of female students significantly declined, whereas there was no self-concept change for male students [34]. A cross-sectional study in a New-Zealand university reported no gender differences in verbal and math self-concept of undergraduate students of the same faculty, suggesting that student's choice of courses was based on confidence and interest in the subject, rather than being determined by the gender stereotype that math is a masculine discipline [35]. Although most studies indicate that female college students have a lower academic self-concept than male college students, more research on self-concept and gender during transition into university is needed.

Studies examining the possible joint effects of autonomous motivation and academic self-concept on academic achievement are scarce. In high school students, evidence was found for a mediating effect of autonomous motivation on the relation between academic self-concept and academic achievement [36]: students perceiving themselves as competent are assumed to be more autonomously motivated which in turn would lead to higher grades. It can be expected that this mediating effect consolidates in later years, during college or university but to our knowledge, this has not been studied so far. As intricate relationships may occur between academic self-concept and autonomous motivation, a person oriented research perspective can be valuable.

### A person oriented research perspective

Former SDT-research has indicated how a person oriented research perspective can allow to categorize individuals in groups with similar motivational characteristics and can increase current understandings on how unique combinations of motivational factors are related to higher or lower levels of academic achievement [13].

Using cluster analyses three different motivational profiles were found in college students [37]: (A) high autonomous and low controlled motivation, (B) high autonomous and high controlled motivation, and (C) low to moderate motivation. Motivational profiles A and B obtained the highest academic achievement scores but students from profile A were more likely to persist in their studies compared to profile B. In another study with college students, also a fourth profile D consisting of students with low autonomous and high controlled motivation was found in addition to the above described three profiles A, B and C [13]. Also in this study, profile A and B had similar learning outcomes measured in terms of cognitive processing, time use and meta-cognitive strategy use. However, students belonging to profile A showed less test anxiety compared to profile B, and slightly higher determination (in terms of higher effort regulation, and less procrastination). Therefore, motivational profile A was associated with a more optimal learning pattern compared to the other groups and hence labeled “good quality of motivation”. In both studies, female students reported a higher autonomous motivation compared to male students [13], [37]. The existence of the same four distinct motivational profiles was confirmed in a study of undergraduate students, and no difference in achievement was observed between motivational profile A (high autonomous motivation, low controlled motivation) and motivational profile B (students with high autonomous and high controlled motivation), suggesting that controlled motivation is not detrimental for study achievement when coupled with high levels of intrinsic motivation [38]. Although it has been suggested that an autonomous motivational profile is more likely to develop in a college or university context, which is often more autonomy supportive compared to the more controlling climate in high school context [9], [37], [39], also university programs require perseverance in some subjects that might not always be perceived as interesting. This could explain why also profiles with high levels of both autonomous and controlled motivation are associated with academic success in college [9]. Taken together, the limited set of person-oriented studies carried out in different educational contexts, show that motivational profiles which are associated with higher autonomous motivation levels are associated with higher academic achievement.

### This study

Previous research has indicated the explanatory value of autonomous motivation and academic self-concept on academic achievement in higher education programs in general and in STEM programs in particular. However, these two important explanatory factors have often been investigated separately [13], [37], [40]. To unravel the joint effects of autonomous motivation and academic self-concept on achievement, a person-oriented research perspective enables to further explore this relationship [10], [11]. The central aims of this study are twofold; (1) to investigate the possible diversity of motivational profiles occurring in first-year STEM students' population and subgroups of individuals based on the relative levels of autonomous motivation and academic self-concept and (2) to examine whether these motivational profiles are differently associated with early academic achievement. Also in addition to previous research, we will draw explicit attention to possible gender differences. Since female college students report higher autonomous motivation [13], [37] and lower academic self-concept [32], [33], we expect that gender differences must be taken into account. It is possible that intermediate profiles might occur, combining a relatively low autonomous motivation with a relatively high academic self-concept on the one hand, in which male students are overrepresented, and a profile with a relatively high autonomous motivation combined with a relatively low academic self-concept on the other hand, in which female students are overrepresented.

The following research questions (RQ) are central in this study:

1. Can different motivation and academic self-concept profiles be discerned among first-year college students in STEM?
2. Is gender associated with motivation and academic self-concept profiles?
3. Are motivation and academic self-concept profiles associated with early academic achievement?

Based upon previous findings from correlation studies investigating either autonomous motivation or academic self-concept in relationship with achievement, we expect that profiles with high levels of autonomous motivation and academic self-concept would be associated with higher academic achievement and *vice versa* (Hypothesis 1). In addition, we expect that students with intermediate motivational profiles, having high levels of only one motivational variable (either autonomous motivation or academic self-concept), would be associated with intermediate levels of academic achievement. Since prior achievement indicators such as high school GPA and ACT scores are strong predictors of university college GPA [7], we control for prior achievement when examining the relation between motivational variables and early academic achievement. Based upon former studies showing gender as an interplaying factor describing student motivation and academic self-concept [13], [37], [40], we expect to observe gender differences in autonomous motivation and academic self-concept profiles, in particular that female students in STEM programs are more autonomously motivated and have a lower academic self-concept compared to male students, resulting in the occurrence of intermediate profiles with an unequal gender distribution (Hypothesis 2). We also expect that the positive effects of a relatively high self-concept on academic achievement are equally important for female and male students (Hypothesis 3) [32], [40].

## Method

### Ethics Statement

Students provided written informed consent for their voluntary participation, and confidentiality was guaranteed. In Belgium, the Law on Experiments on Humans (7 May 2004) obliges ethics approval only for experiments (or studies or research) in which human persons are involved and with the goal of developing knowledge related to health care professions (2004050732/N, Article 2, paragraph 11). The current research is not related to the development of knowledge related to health care professions and is therefore implicitly exempt from ethics approval.

### Sample

The sample consists of first-year (undergraduate) Bachelor students in STEM, participating for the first time to higher education, in a Research University (total student number of 35000 students) in Belgium. All students having followed a secondary education study track are allowed to enroll. Concerning the socio-economic status of the entire student population at this University, the majority of student's parents were professionally active (92% of fathers; 80% of mothers) and had a degree in higher education (67% of fathers; 70% of mothers), 16,5% of the total student population students reported financial problems. The vast majority of first-year college students followed a general secondary education preparing for higher education, but also students from technical and vocational education tracks are allowed to enroll.

### Measures

#### Academic Motivation and Academic Self-concept

To measure students' academic motivation, we used the adapted version of the Academic Self-regulation Scale [13] tapping students' motivation for studying. The questionnaire maps four distinct motivational types, three items of each scale were used. The central proposition was “*I am motivated for my study”* followed by various motives such as *“ because I*'*m highly interested in doing this*” (intrinsic motivation), “[…] *because I want to learn new things*” (identified regulation), “[…] “….*because I want others to think I*'*m smart*” (introjected regulation), “[…] “*because that*'*s what others (e.g. parents, friends) expect me to do*” (external regulation). All 12 items were scored on five-point Likert scales ranging from 1 (*does not apply to me at all*) to 5 (*does apply to me*). Compared to the Academic Motivation Scale [41], the Academic Regulation Scale enables making a more elaborate distinction between autonomous and controlled motivation scales [39].

Students' academic self-concept was tapped by focusing on their academic outcome expectancy in terms of study success. Three items were used in this study about how confident and prepared students felt to succeed in their study: “*I expect that I will be able to succeed in my study*”, “*I feel well prepared for this study*”, and “*I fear this study will be too difficult for me*”. The items were scored on a five-point Likert scale ranging from 1 (*does not apply to me at all*) to 5 (*does apply to me*).

A principal components analysis (PCA) was conducted on the 15 academic motivation and self-concept items with oblique rotation (PROMAX). The Kaiser-Meyer-Olkin measure confirmed adequate sample size, KMO = .74, and all KMO values for individual items were >.63, which is above the acceptable limit of.5 [42]. The initial analysis indicated a clear drop in eigenvalues (i.e., 3.17, 2.65, 1.75, 1.33, 1.04) between the third and fourth factor, and also the scree plot showed an inflection after the third factor. Also, the first three components explained 51% of the variance in the motivation and self-concept items. Taken together, this justified the retention of three components in the analysis. After oblique rotation (PROMAX), the six autonomous motivation items had loadings of at least.40 on the first component, the six controlled motivation items had loadings of at least.58 on the second factor and the three self-concept items had loadings of at least.70 on the third factor. No cross-loadings were found in pattern or structure matrix. Composite scales were created for autonomous and controlled motivation in line with former research [13], by averaging the subscales for intrinsic and identified regulation (autonomous regulation), and for introjected and external regulation (controlled motivation) [13], [43]. The three retained factors had good to average internal consistencies: autonomous motivation (Cronbach α = .77), controlled motivation (Cronbach α = .75) and academic self-concept (Cronbach α = .68). Item composition and distribution format of the composite scales are reported in Table 1.

**Table 1:** Descriptive Statistics of Motivational Scales.

Scale	*N* (items)	*M*	*SD*	Skewness^a^	Kurtosis^b^	*Cronbach α*
Autonomous Motivation	6	3.39	.59	−.05	−.14	.77
Controlled Motivation	6	2.37	.72	.19^c^	−.51^c^	.75
Academic Self-concept	3	3.58	.60	−.16^c^	−.07	.68

*Note*. N = 1473, values for all scales range 1–5 (Likert).

a Critical value: 2*standard error of skewness  =  |.13|.

b Critical value: 2*standard error of kurtosis  =  |.25|.

c Exceeds the corresponding critical value.

#### Prior Study track & Achievement

Students' prior achievement was an overall percentage provided by the student and labeled as “*prior high school result*”. Of the respondents, 89% reported this percentage. The majority of the students followed a general secondary education track with six weekly lessons of mathematics or more (information obtained from university records). These so-called “traditional” tracks were Mathematics & Science, Latin & Mathematics, Latin & Science, Greek & Mathematics, Greek & Science. All other prior study tracks were labeled as non-traditional, some examples are Human Science, Technical Science, Modern languages & Science.

#### Early academic achievement

Students' academic achievement data were obtained from the university database after the first exam period in January. Two measures were used: the overall study result after the first exam period (mean of scores on all exams, expressed as percentage) and the percentage of credits obtained (amount of ECTS study points that a student has earned during a particular time period, compared to the total amount of credits that were attempted during that period, expressed as percentage – *e.g.* when a full time student passes on 24 credits of the 30 credits taken during the first semester, he has 80% of his credits obtained).

### Plan of analyses

1) Descriptive statistics: scale means and standard deviations were calculated within the total sample and within subgroups (gender, prior study track). Means and correlations were calculated for those students for which variables were available. Significance of differences in mean scores by gender and prior study track was investigated by means of independent t-tests, post-hoc tests and calculation of effect sizes. Cut-off criteria of Cohen's *d* [44] were used, in which *d* = .2 is indicative of a small effect while *d* = .5 and *d* = .8 represent a medium and large effect respectively. 2) Pearson correlations were calculated to explore the relationship within and between the motivational variables and variables related to prior and early academic achievement. Criteria of Cohen [44] were applied to interpret the strength of the correlation patterns, in which *r*>.10 and <.30 is indicative for a weak, *r*>. 30 and <.50 for a moderate and *r*>.50 for a strong correlation.3) Cluster analyses on autonomous motivation and academic self-concept were carried out to distinguish motivational profiles among student groups. A two-step procedure was used, with a hierarchical clustering procedure (Ward's method) to determine the optimal cluster number and initial seeds for a second non-hierarchical k-means clustering procedure, as described in [13]. A cluster solution will be retained in the analyses if the variance in the constituting dimensions is above the 50% threshold [43]. To examine the stability of a cluster solution, the double-split cross-validation procedure was used [13]. 4) Variance analyses and calculation of effect sizes was carried out to explore the relationship between the obtained cluster solution, early academic achievement and gender.

## Results

### Participants

Data of two cohorts of first-year students were collected at the beginning of academic year 2009–2010 (cohort 1) and 2010–2011 (cohort 2). First-year students enrolling in university's undergraduate STEM programs were during a class period asked to fill out a questionnaire. Of the total group of 1,673 students, 1,480 filled out the questionnaire and provided informed consent (response rate 88%). Of the participating students, 23% was female, which is comparable to the average participation of first year female students to the corresponding STEM undergraduate study fields (applied sciences, sciences, applied biosciences) in Flanders (Dutch speaking part of Belgium) [45]. The gender distribution varied depending on the chosen study program, and the percentages of female students in a class group ranged from 6% in “Exact Sciences: Computer Science” (66 students), 14% in “Engineering Science” (800 students), 27% in “Exact Sciences: Mathematics/Physics” (115 students), 34% in “Exact Sciences: Chemistry/Biochemistry/Biology/Geology/Geography” (303 students) to 44% in “Bioscience Engineering” (196 students).

Although t-testing revealed slight differences in mean levels of autonomous motivation and academic self-concept between both cohorts which might be due to the sample size, a regression analysis with early academic achievement as dependent variable and the measured variables described below as independent variables (Model 1, Adjusted R Square  = .371), showed that the addition of the dummy variable “belonging to cohort 1” in Model 2 did not have a significant effect on achievement (Model 2, Adjusted R Square  = .371, *β* cohort 1 = .031, *p* = 0.189). Therefore, both cohorts were analyzed together. The total sample consisted of 1,480 students, i.e. 1,143 male students and 337 female students, with an average age of 17.6 years.

### Descriptive statistics

Table 1 and 2 show the descriptive statistics and gender differences of all the variables used in this study, for male (N = 1,143) and female (N = 337) students in STEM. Female students reported a slightly higher prior high school result, a higher autonomous motivation, and a lower academic self-concept compared to male students. There was no difference in controlled motivation between male and female students. Early academic achievement scores of female students were slightly higher than of male students. Based on Cohen's convention [44] for effect size *d*, the effect size of all group mean differences was small, except for autonomous motivation (*d* = .45) which can be considered as a medium effect.

**Table 2:** Gender Differences for Measured Variables of STEM Students.

Variable	Male *M (SD)*	*Female M (SD)*	*M* (difference)	*t*	*df*	*p (2-tailed)*	*d*
Prior high school result^a^	73.53 (7.07)	74.89 (6.46)	−1.36	−3.147	1324	.002	.20
Autonomous Motivation^b^	3.32 (0.59)	3.58 (0.56)	−0.26	−7.185	1486	<.001	.45
Controlled Motivation^b^	2.38 (0.72)	2.37 (0.73)	0.01	0.014	1486	.888	.01
Academic Self-concept^b^	3.61 (0.61)	3.43 (0.60)	0.17	4.546	1486	<.001	.30
Overall Academic exam result^a^	47.10 (17.88)	50.56 (16.27)	−3.45	−3.357	1474	.001	.20
Academic credits obtained^a^	52.16 (36.13)	57.16 (35.13)	−5.00	−2.278	1468	.023	.14

a expressed as %.

b expressed on 5 point Likert scale.

Table 3 shows the descriptive statistics of the variables used in this study, for students with a traditional prior study track (N = 1,270), versus students with a non-traditional prior study track (N = 210). Traditional students reported a higher prior high school result, a lower autonomous motivation, a higher controlled motivation, and a higher academic self-concept compared to non-traditional students. Early academic achievement scores of traditional students were significantly higher than the scores obtained of non-traditional students. The effect size (Cohen's *d)* of all group mean differences was small to medium, except for overall academic exam result (*d* = .82) and academic credits obtained (*d* = .72) which can be considered as a large effect.

**Table 3:** Prior Traditional vs Non-traditional Study Track Differences for Measured Variables of STEM Students.

Variable	traditional *M (SD)*	non-traditional *M (SD)*	*M*(difference)	*t*	*df*	*p (2-tailed)*	*d*
Prior high school result^a^	74.05 (6.92)	72.46 (6.90)	1.58	2.841	1318	.005	.23
Autonomous Motivation^b^	3.35 (0.58)	3.52 (0.62)	−1.17	−3.902	1478	<.001	.28
Controlled Motivation^b^	2.39 (0.73)	2.25 (0.67)	0.15	2.773	1478	.006	.20
Academic Self-concept^b^	3.60 (0.59)	3.41 (0.61)	0.18	4.449	1478	<.001	.32
Overall academic exam result^a^	49.83 (16.92)	36.04 (16.76)	13.79	10.833	1466	<.001	.82
Academic credits obtained^a^	56.68 (35.30)	32.54 (32.90)	24.137	9.663	1468	<.001	.72

a expressed as %.

b expressed on 5 point Likert scale.

### Relating autonomous motivation, academic self-concept, and early academic achievement

Table 4, left panel shows the bivariate correlations (Pearson correlation coefficients) between the variables used in this study, computed for the entire group of STEM students. As expected, there is a moderate positive correlation between prior high school result and early academic achievement (i.e., overall academic exam result and academic obtained credits). A small but significant positive correlation exists between academic self-concept and autonomous motivation, between autonomous motivation and early academic achievement, and between academic self-concept and early academic achievement respectively. There is no correlation between controlled motivation and achievement indicators, and a small negative correlation between controlled motivation and academic self-concept.

**Table 4:** Bivariate Correlations for all STEM students (left), Male STEM Students (Middle) and Female STEM Students (Right).

		All students				Male students					Female students				
	1	2	3	4	5	1	2	3	4	5	1	2	3	4	5
1 Prior high school result	-					-					-				
2 Autonomous Motivation	.15***	-				.13***	-				.18**	-			
3 Controlled Motivation	.00*^ns^*	*−.*05*^ns^*	-			*−.*02*^ns^*	*−.*05*^ns^*	-			.07*^ns^*	*−.*06*^ns^*	-		
4 Academic Self-concept	.11***	.15***	−.09**	-		.13***	.20***	−.07*	-		.06*^ns^*	.09*^ns^*	−.14*	-	
5 Overall academic exam result	.48***	.10***	.03*^ns^*	.25***	-	.50***	.10**	.03*^ns^*	.27***	-	.38***	.05*^ns^*	.05*^ns^*	.22***	-
6 Academic credits obtained	.44***	.08**	.04*^ns^*	.21***	0.90***	.45***	.07*	.04*^ns^*	.23***	.90***	.36***	.06*^ns^*	.05*^ns^*	.19***	.90***

***p<.001,

**p<.01,

*p<.05, *ns*  =  non significant.

N of students range; all: 1320–1480; males: 1015–1134; females: 294–336.

Since the descriptive statistics in Table 2 show significant differences in autonomous motivation and self-concept for male and female students, we performed a gender-specific correlation analysis for the included variables and significant gender differences are found (Table 4, middle and right panel). The correlation pattern of male students (Table 4, middle panel) is comparable to the correlation pattern of the entire group of STEM students (Table 4, left panel), which is not surprising since 77% of the population is male. Female students (Table 4, right panel) display a slightly different pattern: there is no significant correlation between prior high school result and academic self-concept, between autonomous motivation and academic self-concept, nor between autonomous motivation and early academic achievement. Both male and female students have a small positive correlation between academic self-concept and early academic achievement.

### Motivational profiles of first-year STEM students

In order to explore the presence of motivational profiles within the sample of STEM students, we performed a cluster analysis on the two motivational dimensions showing a positive correlation with academic achievement, namely autonomous motivation and academic self-concept. Since there was no significant correlation between controlled motivation and academic achievement in our population, the controlled motivational dimension was not included in further analyses.

Before applying Ward's method, we calculated z-scores for autonomous motivation and academic self-concept, and removed seven univariate outliers. A four-cluster solution in the resulting sample of 1,473 students, explained most of the variance in the constituting dimensions, namely 57% of the variance in autonomous motivation and 63% of the variance in academic self-concept, both of which are above the 50% threshold [46]. In a three-cluster solution, the explanatory power across the substituting dimensions was lower (55% for autonomous motivation and 51% of the variance in academic self-concept), and a two-cluster solution was not retained because the explanatory power was below the 50% threshold.

**Figure 1:**
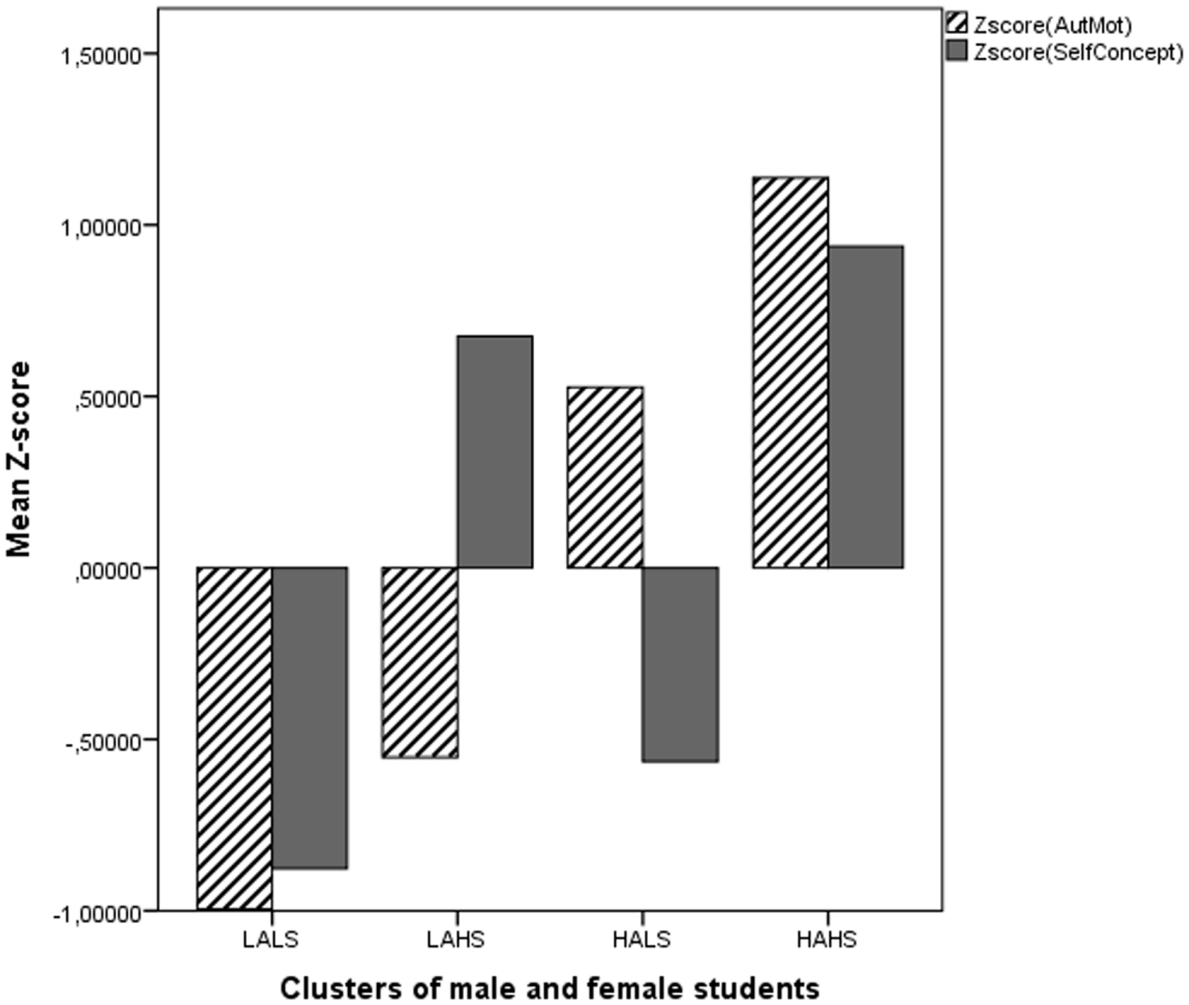
Autonomous motivation and academic self-concept of four-cluster solution of entire group of STEM students. Average z-scores for autonomous motivation (striped bar) and academic self-concept (gray bar) of the four-cluster solution of both male and female STEM students together.

In a second step, the four-cluster centers extracted with Ward's method were used as initial seeds in a k-means clustering procedure. The average z-scores of the final cluster solution are depicted in Figure 1. The following groups can be discerned: (LA-LS) students scoring low both on autonomous motivation and academic self-concept (n = 313, 21%), (LA-HS) students with a low level of autonomous motivation and a high academic self-concept (n = 429, 29%), (HA-LS) students with a high level of autonomous motivation and a low academic self-concept (n = 438, 30%), and (HA-HS) students scoring high both on autonomous motivation and academic self-concept (n = 293, 20%).

When the average z-scores of the two clusters having high levels of autonomous motivation are compared, it is interesting to note that the cluster with high levels of both dimensions (HA-HS) has a significantly greater level of autonomous motivation than the cluster with the mixed profile (HA-LS) (mean z-score on autonomous motivation is 1.14 in HA-HS versus 0.53 in HA-LS, *p*<.001). Accordingly, the cluster with low levels of both dimensions (LA-LS) has a significantly lower level of autonomous motivation compared to the cluster with the mixed profile (LA-HS) (mean z-score on autonomous motivation is −1.00 in LA-LS versus −0.55 in LA-HS, *p*<.001). The same is true when the average z-scores of the two clusters having high levels of academic self-concept are compared: the cluster with high levels of both dimensions (HA-HS) has a significantly greater level of academic self-concept compared to the cluster with the mixed profile (LA-HS) (mean z-score on self-concept is 0.94 in HA-HS versus 0.68 in LA-HS, *p*<.001); and the cluster with low levels of both dimensions (LA-LS) has a significantly lower level of academic self-concept than the cluster with the mixed profile (HA-LS) (mean z-score on self-concept is −0.88 in LA-LS versus -0.56 in HA-LS, *p*<.001).

The average kappa value of the four-cluster subsamples (.74) shows a good level of agreement and provides evidence for the internal stability of the four-cluster solution.

### Relating student characteristics to motivational profiles

A chi-square test of independence to examine the relation between cluster and sample characteristics such as prior study track, current STEM program and gender showed significant dependence for all variables tested.

Students from a prior study track with less than 6 weekly lessons of mathematics were overrepresented in the high motivation/low self-concept cluster (HA-LS), whereas students from a prior study track with 6 or more weekly lessons of mathematics were overrepresented in the low motivation/high self-concept cluster (LA-HS) (*χ^2^* (3, *N* = 1473)  = 27.85, *p*<.001), Apparently, a typical “cross” profile is associated with the level of mathematics in prior study track: students from a low math prior study program, have a relatively high level of autonomous motivation, but have a low academic self-concept when they start their academic STEM study demanding a high mathematics preparation, whereas students from a high math profile, have a higher academic self-concept but are relatively low in autonomous motivation.

Engineering Science students were overrepresented in the LA-LS cluster and underrepresented in the HA-HS cluster, whereas Exact Science students were overrepresented in the HA-HS cluster and underrepresented in the LA-HS cluster (*χ^2^* (6, *N* = 1473)  = 44.74, *p*<.001). Exact Science programs that are characterized by the fundamental study of one discipline attract more students with a high level of both motivation and self-concept compared to the more application-oriented Engineering Science programs.

Consistent with the observed gender differences in autonomous motivation and self-concept, chi-square testing revealed a significant cluster assignment × gender effect (*χ^2^* (3, N = 1473)  = 54.17; *p*<.001). Female students were underrepresented in the low motivation/high self-concept cluster (LA-HS) and overrepresented in the high motivation/low self-concept cluster (HA-LS) (Table 5).

**Table 5:** Gender-specific Percent Distribution over the four Clusters.

	Gender	
	Male (N = 1137)	Female (N = 336)
LA-LS	23% (2.8)	16% (−2.8)
LA-HS	32%(5.0)	18%(−5.0)
HA-LS	26%(−6.4)	44%(6.4)
HA-HS	19% (−1.3)	22%(1.3)

*Note*. Adjusted standardized residuals appear in parentheses after the observed percentages.

Given these significant gender differences, we examined whether the same motivational profiles would appear when male and female students were analyzed separately, by performing an additional series of cluster analyses on both samples independently.

**Figure 2:**
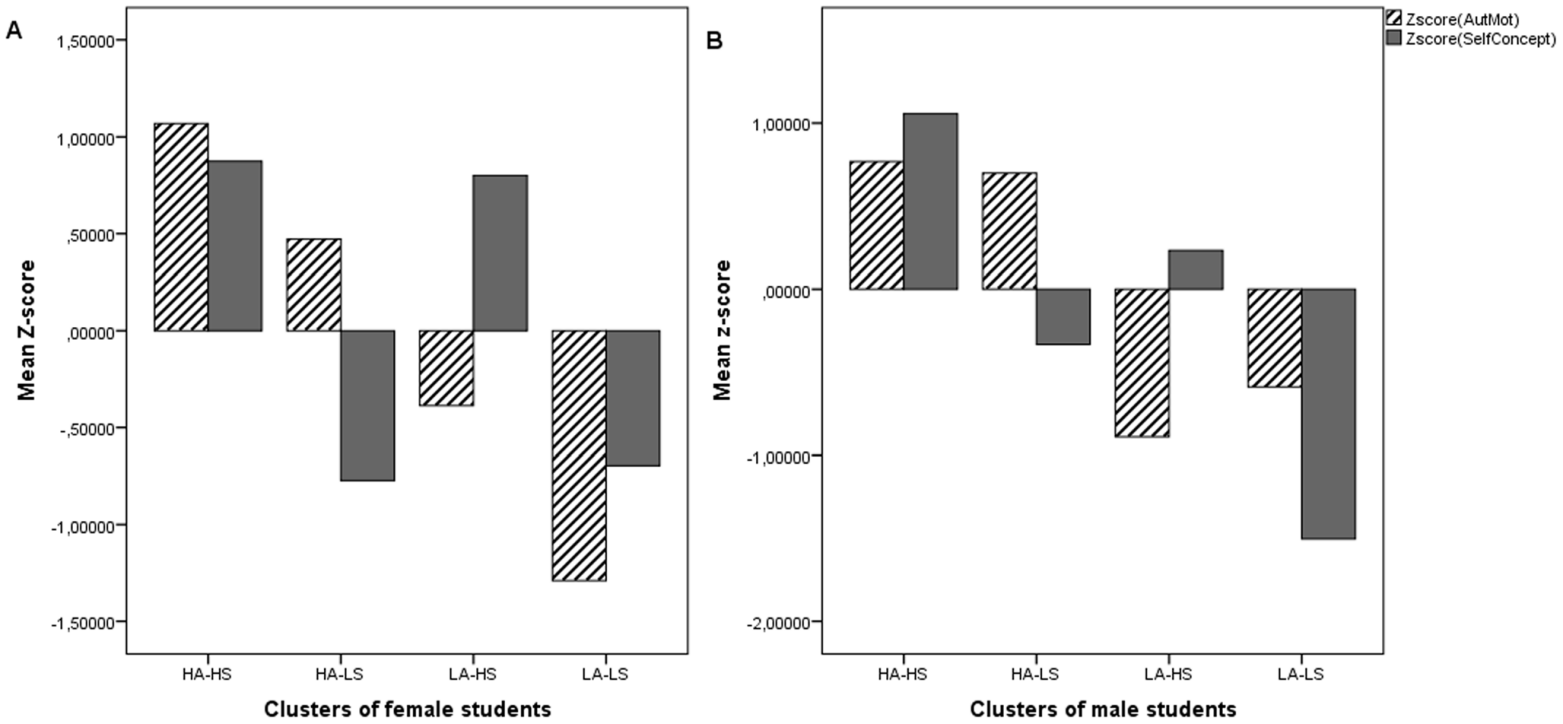
Autonomous motivation and academic self-concept of four-cluster solution of male and female STEM students separately. Average gender-specific z-scores for autonomous motivation (striped bar) and academic self-concept (gray bar) of the four-cluster solution of female (panel A) and male (panel B) STEM students separately.

The four-cluster solution for female students explained 64% respectively 59% of the variance in the constituting dimensions autonomous motivation and academic self-concept, for male students this was 52% respectively 70%. A three- or two-cluster solution had less explanatory power for both groups. The average z-scores of the four final cluster solution for male and female students are depicted in Figure 2.

Both for male and female students, an analysis of variance and post hoc comparisons (Tukey HSD) showed that cluster means of the constituting dimensions were significantly different. F-values and mean values of the constituting dimensions for male and female students are shown in Table 6.

**Table 6:** Mean Values of the Constituting Dimensions and of Outcome Variables for the four Extracted Clusters of Male and Female Students.

	LA-LS	LA-HS	HA-LS	HA-HS	*F*	*η^2^*
**Male students**	N = 169	N = 327	N = 262	N = 257		
*Constituting Dimensions*						
Autonomous motivation	2.99_b_	2.80_a_	3.73_c_	3.77_c_	*F* (3, 1136) = 535.73, *p*<.001	
Academic Self-concept	2.74_a_	3.75_c_	3.41_b_	4.25_d_	*F* (3, 1136) = 880.39, *p*<.001	
*Outcome Variables*						
Overall academic exam result	41.2_a_	48.8_b_	46.5_b_	51.6_c_	*F* (5, 1010) = 18.46, *p*<.001	.05
Academic credits obtained	42.4_a_	56.0_b_	49.0_a_	60.0_b_	*F* (5, 1010) = 13.32, *p*<.001	.04
**Female students**	N = 58	N = 85	N = 93	N = 58		
*Constituting Dimensions*						
Autonomous motivation	2.90_a_	3.39_b_	3.85_c_	4.17_d_	*F* (3, 335) = 241.15, *p*<.001	
Academic Self-concept	3.05_a_	3.91_b_	3.02_a_	3.96_b_	*F* (3, 335) = 186.24, *p*<.001	
*Outcome Variables*						
Overall academic exam result	49.7_a_	53.6_a_	50.2_a_	52.9_a_	*F* (5, 293) = 1.31, *p* = .27	.01
Academic credits obtained	57.9_a_	63.4_a_	54.8_a_	61.6_a_	*F* (5, 293) = 1.21 *p* = .31	.01

a mean values with different subscripts were significantly different at the.001 level (post hoc Tukey LSD).

b univariate F-values, partial *η* ^2^ and pairwise comparisons of estimated means (overall academic exam result, percentage of credits obtained) are shown, estimated means with different subscripts were significantly different at least at the.05 level (post hoc Tukey). The covariates were evaluated at the following values for male students: prior high school result  = 73.5, prior study track  = .14, for female students: prior high school result  = 74.84, prior study track  = .11.

Thus, each student had two cluster assignments to one of four possible clusters: (1) a general cluster assignment based on the entire sample of male and female students, (2) a gender-specific cluster assignment calculated for males or females separately. The latter cluster assignment has the advantage that the effect of gender-specific differences in motivational constructs is minimized.

The convergence between the general and gender-specific cluster assignments was acceptable (average kappa value κ = .60 for male students, κ = .74 for female students), and supports the internal stability of the four-cluster solution. Although the convergence between both cluster assignments is sufficient, we will use only the gender-specific cluster assignments in subsequent analyses, since this allows us to focus on achievement differences between the various motivational profiles independent of possible gender effects.

### Motivational profiles and early academic achievement

We examined whether differences in early academic achievement are associated with different motivational profiles of male and female students. Since there is an effect of prior high school result and of prior study track on academic achievement, these variables were added as covariates to the analyses.

The effect of cluster membership on study outcome was studied for male and for female students separately, by means of univariate analyses of variance and post hoc comparisons (Tukey HSD), with “cluster membership” as independent variable, “academic achievement” (academic credits obtained) as dependent variable, and “prior high school result” and “prior study track” as covariate.

For male students, the covariates “prior high school result” and “prior study track” were significantly related to the amount of credits obtained, with F (1, 1011)  = 242.52, *p*<.001, *r* = .44 for “prior high school result”, and *F* (1, 1011)  = 49.60, *p*<.001, *r* = .22 for “prior study track.” (Table 6).

There was a significant effect of cluster assignment on amount of credits obtained, after controlling for “prior high school result” and “prior study track,”, *F* (3, 1011)  = 13.32, *p*<.001, partial *η^2^* = .04.

Planned comparisons showed that male students with high autonomous motivation and a high academic self-concept (HA-HS) obtained significantly more academic credits compared to male student with low autonomous motivation and low academic self-concept (LA-LS), *t* (1011)  = −5.69, *p*<.001, *r* = .18, and compared to male students with high autonomous motivation and a low academic self-concept (HA-LS), *t* (1011)  = −4.09, *p*<.001, r = .13.

A similar pattern was observed when overall academic exam result was used as indicator of early academic achievement (Table 6).

Pairwise comparisons of estimated means (overall exam result, credits obtained) of male and female students, controlled for by “prior high school result” and “prior study track” are shown in Table 6.

Male students from the LA-LS cluster have the lowest mean academic achievement scores, both when measured as overall academic exam result and as percentage of academic credits obtained. Students from the HA-HS cluster have the highest mean academic achievement. Male students with a high self-concept but low autonomous motivation (LA-HS), still have a slightly higher mean academic achievement than male students with a high autonomous motivation and low self-concept (HA-LS).

At first sight, autonomous motivation seems to be positively assocciated with early academic achievement, since male students with a high autonomous motivation but low self-concept (HA-LS), perform better than students with a low autonomous motivation and a low academic self-concept (LA-LS), and also male students with a high autonomous motivation and high self-concept (HA-HS), perform better than students with a low autonomous motivation and a high academic self-concept (LA-HS). However, it should be noted that not only the ratio of autonomous motivation and self-concept is different between the LA-LS and HA-LS group, but also the level of self-concept: the mean level of academic self-concept in the LA-LS group is significantly lower than the mean level of academic self-concept in the HA-LS group (Table 6). Therefore, the positive relationship between autonomous motivation and early academic achievement might be also mediated by the level of academic-self-concept.

Also for female students, the covariates “prior high school result” and “prior study track” were significantly related to the amount of credits obtained, with F (1, 293)  = 42.43, *p*<.001, *r* = .36 for “prior high school result”, and F (1, 293)  = 17.32, *p*<.001, *r* = .24 for “prior study track.”

However, in contrast to the significant cluster effects on early academic achievement observed with male students, there was no significant effect of cluster assignment on early academic achievement measured as academic credits obtained, after controlling for “prior high school result” and “prior study track,” F (3, 293)  = 1.21, *p* = .31, partial *η^2^* = .01. The same results were obtained when overall academic exam result was used as measure for academic achievement, F (3, 293)  = 1.31, *p* = .27, partial *η* ^2^ = .01.

Pairwise comparisons of estimated means (overall academic exam result, academic credits obtained) of female students, controlled for “prior high school result” and “prior study track” are shown in Table 6.

For female STEM students, motivational factors do not seem to have a positive effect on early achievement, after controlling for “prior high school result” and “prior study track.”

When the mean values of early academic achievement are compared for male and female students of the same motivational profile, it is striking that female students with a low autonomous motivation and a low self-concept (LA-LS), have a significantly higher mean academic achievement compared to male students with a low autonomous motivation and a low academic self-concept (LA-LS) (mean exam result of female LA-LS students  = 48.53% compared to 41.57% for male LA-LS students, *p*<.01, F(7, 1300)  = 9.10, covariates evaluated at the following values: “prior high school result”  = 73.83, “prior study track”  = .13.)

## Discussion and Conclusion

Previous studies have convincingly shown the explanatory value of either autonomous motivation or academic self-concept regarding academic achievement. In this study, a person-oriented research perspective was used taking into account both autonomous motivation and academic self-concept, to provide first evidence of the presence of various motivational profiles among STEM students, their distribution across gender and specific student groups and to explore associations with early academic achievement.

In contrast with former research, explorative correlation analysis showed no significant association between academic self-concept and academic motivation for female students in our sample. Both motivational variables were measured during the first week of the academic year, in full transition from secondary school to the new environment of university. Since in secondary schools female students express a lower interest in STEM compared to male students [47], we can expect that the minority of females that eventually choose to study STEM in university are highly motivated. But in contrast to secondary school, where the gender composition of most classes is more or less balanced, it is possible that during the first weeks of the academic year and the first confrontations with large and predominantly male classes, their minority status becomes really apparent and affects their academic self-concept. Jackson et al. [34] reported that during transition from secondary school to university, self-concept of female students declines more than self-concept of male students and that female students are more susceptible to the Big-Fish-Little-Pond effect (BFLPE) [48]. This transition effect might become aggravated by the minority status of female students in STEM programs, and as a result, it might influence the association between motivation and self-concept.

In order to gain a more comprehensive picture of the possible synergy of both motivational variables in university STEM programs, we used a person-centered analysis approach.

### Motivational profiles

Both for male and female students four different motivational profiles can be detected based on the ratio of autonomous motivation (A) and the academic self-concept (S): students who score high (HA-HS) respectively low (LA-LS) on both dimensions, and students who score high on one dimension and low on the other (HA-LS and LA-HS). There was a significant effect of gender on cluster distribution, with female students being overrepresented in the HA-LS group, and male students being overrepresented in the LA-HS group. This effect can be attributed to the higher level of autonomous motivation and the lower level of academic self-concept of female students compared to male students. Also in previous person-oriented studies examining motivational profiles based on autonomous and controlled motivation levels [13], [18], [40], female students are overrepresented in the clusters with high levels of autonomous motivation. In our study, the unbalanced gender distribution over the clusters is even more pronounced, since gender differences not only occur in levels of autonomous motivation, but also in levels of academic self-concept. The measured academic self-concept in this study can be considered as a mathematics academic self-concept, since the academic self-concept was explicitly related to the expectancy of success in a particular STEM study program, which is generally known to require high mathematics entrance levels. As mentioned before, male students in general report higher mathematics academic self-concept than female students [32], [33], so the lower academic self-concept of female students compared to male students, fits in this observation.

Given this pronounced gender differences in both motivational dimensions, all students were reassigned to clusters in separate cluster analyses for female and male students, and the subsequent analyses were based on these gender-specific clusters. Both for male and female students a four-cluster solution is the most stable solution, with the same relative ratios between autonomous motivation and academic self-concept occurring in both gender groups, although the mean levels of the motivational constructs differ between the gender groups.

### Early Academic achievement

Previous research pointed out that gender is an important variable to be taken into account to better understand differences in student achievement in first-year higher education in general, [7], [33], [34] and in STEM programs in particular [33] and this study provides further insights. The person-oriented research perspective allowed us to focus on academic achievement differences between the various motivational profiles within both gender groups.

For male students, different early achievement outcomes were associated with the different motivational profiles, after controlling for prior achievement and prior study track. Male students with high levels on both motivational dimensions had a significantly better achievement compared to male students with low levels on both dimensions. These findings matched the expectations about the explanatory value of academic self-concept and autonomous motivation regarding academic achievement [9], [21], [30], but have now also been found within specific student groups characterized by both motivational characteristics. Moreover, when the mean levels of academic self-concept in the male clusters are taken into account, it seems that academic self-concept is more important for academic achievement than autonomous motivation. In the preparatory steps of this study, we also found stronger correlations between academic self-concept and achievement than between autonomous motivation and achievement. Marsh et al. [40] had similar observations and demonstrated that prior academic self-concept predicts subsequent academic achievement, beyond what can be explained in terms of academic interest.

Contrary to the expectations, female STEM student's motivational factors were not found consistently related with prior high school results, nor with early academic achievement after controlling for prior achievement and prior study track.

The absence of a significant correlation between prior high school result and academic self-concept for female students might be related to the particular context of female students in transition from secondary school to university STEM programs. Most self-concept studies have studied the relation between achievement and self-concept in longitudinal studies in primary or secondary education, in order to elucidate the causal relation between both variables (self-enhancement model, skill development model, reciprocal effects model, see [27]) and possible developmental effects. In such longitudinal studies, pupils are studied while they progress from one grade to another, but they remain in the same school and education type. In this context, a closer association between achievement and self-concept can be expected, although this is not always the case and gender differences have been described: a five wave-longitudinal research in Belgian secondary education found no evidence for self-enhancement or reciprocal effects model for female students [49]. When students go through transitions between different education types (*eg*. from secondary to higher education), it can be expected that associations between prior achievement in secondary education, and self-concept at the start of university might weaken. Such a transition requires adolescents to adjust to a new environment with challenging demands, and might result in feelings of uncertainty and a declined self-concept, especially for female students [34]. It can be expected that the absence of central exams or an admission procedure, as is the case in Belgian higher education, might increase uncertainty for some student groups who already have a lower self-concept, such as female students. Taking into account the context of the study, the observed absence of correlation between prior achievement and self-concept of female students underlines the necessity for more detailed investigations about the evolution in motivational variables during transition, taking into account gender and context characteristics. Especially in the contexts of STEM education on higher education level further research is needed.

Also the absence of a significant association between autonomous motivation and subsequent early academic achievement for female students is intriguing. A possible explanation might be related to the minority status of female students in STEM university programs. This might lead to stereotype threat [50], increased pressure and thus also to higher levels of controlled motivation that would possibly counteract the beneficial effects of autonomous motivation. However, the absence of differences in controlled motivation between male and female students does not point towards a negative effect of controlled motivation on autonomous motivation. Another possibility is that stereotype threat experienced during the semester could cause a more rapid decline in autonomous motivation levels of female students. In this study, motivation was measured at one time point only, the very beginning of the academic year, so we can not speculate on changes in motivation levels during the semester. Although a longitudinal study on the effects of stereotype threat experienced by female college students in biology and biochemistry courses did not show an effect of prior stereotype endorsement on subsequent autonomous motivation [51], more longitudinal research is needed to investigate possible context effects on shifts in motivation and academic self-concept levels of college STEM students, also in more ‘masculine’ courses like physics, mathematics and engineering.

Another explanation may be that more subtle gender differences must be taken into account, such as social desirability bias affecting female reporting behavior. Although in ethics research, it was shown that this bias confounded observed gender differences [52], to our knowledge, no gender sensitive models of student motivation have been developed within existing SDT research or research on academic self-concept. This facet might be important to explore in future motivation studies in STEM education.

Most of motivational research has focused on primary and secondary education students, while the few studies on higher education students were mostly in gender balanced or female dominated study programs such as educational or psychological sciences. It is possible that the female students in STEM have higher levels of motivation compared to female students in non-STEM, since they made a gender-atypical study choice. It might be possible that the absence of a significant effect might be due to ceiling effects, but this has to be further examined in additional studies with increased levels of sample size for male and female students.

Another optional explanation might be related to the fact that our study took place in Belgium, on STEM students during the transition to university. The Belgian context is particular since entry to almost all university programs (including STEM programs) is free to all K12 students leaving secondary education. However, the variance between school levels, study tracks and individual students is high, and there is no central exam at the end of secondary education. Although most incoming students are aware that STEM programs require prior mathematics knowledge, they have no objective information on whether their individual mathematical knowledge and skills are sufficient to start a STEM program, and they are not able to gauge how “good” they are compared to other STEM students. Thus, also the BFLPE [48] might influence the experience of incoming (female) students during transition, and this particular context might cause a greater variation in motivational variables, and result in a decreased association with achievement for particular students.

The fact that real grades and not standardized tests were used as achievement indicators might also contribute to the observed small (for male) and absent (for female) association between motivation and achievement: it has been suggested [53] that high stakes school grades also reflect motivational properties, in contrast to low stakes standardized tests. Thus, it is possible that this effect is greater for female students due to their higher motivation levels, and that the absence of significant effects of motivational constructs for female students, after controlling for prior school grades, is related to the use of this achievement indicator.

The above mentioned possible explanations are all hypotheses that must be further examined in future research. It does show, however, that the interplay between motivation, gender and cognitive variables in STEM programs, is complex and should also take into account other contextual variables.

### Limitations and research perspectives

This study focused on motivational profiles and gender differences in STEM programs in general, but abstraction was made of the differences existing between various programs of study in STEM with varying percentages of female students. For example in Bioscience Engineering, female students comprise 44% of the student population, whereas in Engineering Science only 14% of the students are female. It can be expected that also these different contexts influence the motivational profiles and further research should take this into account.

The prime interest of this study was to explore the relationship between various motivational profiles and academic achievement, controlling for important explanatory variables, such as gender, prior achievement and prior study track [7]. In this study we were not able to control for other background characteristics that are known to have a smaller effect on academic achievement in college [7], such as socio-economic status, parents' level of education or being first generation college attendees.

Motivation and academic self-concept were measured only once, at the beginning of the academic year. During the first semester, adjustments of motivation and academic self-concept levels could occur, which might result in shifts in cluster assignment. The cross sectional nature of our study did not allow to infer a causal link between particular motivational profiles and academic achievement but it is important to investigate this further. Especially longitudinal research designs may provide further insight into the stability or variability of motivational profiles during the first year and the association with drop-out, long-term academic achievement, and retention.

Also, including amotivation as motivational variable could possibly shed further light on the presence of different motivational profiles in STEM education. It might also be an important variable regarding the further identification of motivational profiles which can be called to be more ‘at risk’, since amotivation has been found associated with more negative learning outcomes in first-year higher education [54].

### Implications for practice

This study identified that some male student groups are more at risk than others, based on their motivational profiles. Male students scoring low on both motivational dimensions might benefit from interventions focused on increasing both their motivation and academic self-concept. In particular academic self-concept is an essential prerequisite for successful studies in STEM. Even though academic self-concept of freshman students entering higher education is necessarily based on previous high school success [55] and might not always be very accurately measured, it still is a better predictor of academic achievement than the level of autonomous motivation, even for female students. Students with low initial levels of autonomous motivation in combination with low academic self-concept are at risk for procrastination behavior when they are confronted with the high demands of STEM studies in the first months of the academic year [13], [56], [57]. Therefore, it would be useful to provide these students early on with explicit feedback on their motivational drive and academic self-concept regarding their study, complemented with content-specific support and training in study and time management skills [58]. In individual counseling programs, tutors should be aware of the lower academic self-concept of female students in general, and adjust their feedback accordingly. Female students in STEM programs might benefit more from encouragement and positive feedback aimed at increasing their academic self-concept. Regardless the above made speculations which can be made on how to foster students' motivation and academic-self-concept, different motivational profiles are present in university STEM programs both in male and female student groups. This finding also suggests that not all student groups will have the same needs of support or guidance. A more differentiated view on student guidance, taking into account these differences in motivational profiles, seems a valuable path to explore further.
